# CyDENT: A tale of CRISPR-free strand-specific base editor for nuclear and organellar genomes

**DOI:** 10.1016/j.omtn.2025.102754

**Published:** 2025-11-11

**Authors:** Mahnoor Jamil, Ambreen Zahra, Rizwana Maqbool, Muhammad Imran Arshad, Sultan Habibullah Khan

**Affiliations:** 1 Center for Advanced Studies in Agriculture and Food Security, University of Agriculture, Faisalabad, Pakistan; 2 Center of Agricultural Biochemistry and Biotechnology, University of Agriculture, Faisalabad, Pakistan; 3 Department of Plant Breeding and Genetics, University of Agriculture, Faisalabad, Pakistan; 4 Department of Epidemiology and Public Health, University of Agriculture, Faisalabad, Pakistan; 5 Public Health Department, College of Applied Medical Sciences, Qassim University, Al-Qassim, Saudi Arabia

## Main text

Base editing is a promising technique for introducing changes in DNA at single-nucleotide resolution. However, CRISPR-Cas9 base editors and TALE (transcription activator-like effector)-mediated base editors (DddA-derived cytosine base editor [DdCBE] and TALE-linked deaminase [TALED]) have been reported to have certain limitations, namely the inability to target organellar genomes and mutations in the non-target strand of DNA, respectively. Recently, a new base editing tool, cytidine deaminase-exonuclease-nickase-TALE (CyDENT), has offered the opportunity to target an organelle’s genome in a strand-specific manner. This base editor holds great potential in human disease therapeutics and can also be extended to the development of crop varieties with improved agronomic traits by introducing base substitutions.

### TALE and CRISPR-mediated base editors

Genome editing enables scientists to precisely edit the DNA in a targeted and sequence-specific manner. Genome editing has rapidly become a preferred tool for genetic tweaking as well as for its applications in therapeutics and diagnostics. The scientific advancements in the field of genome editing have introduced various genome editing modules, i.e., nucleases, transposases/recombinases, base editors (CRISPR and TALE-based), and prime editors (only CRISPR-based reported yet), that have driven unmatched progress in life sciences owing to the unstoppable discovery of their new and improved versions, e.g., BE3, BE4, ABE7.10, DdCBE, and TALED with greater editing efficiencies.^1^^,^^2^ Among the aforementioned editing modules, base editing provides more precise control to introduce mutations at a single base resolution.^3^ Both CRISPR-Cas and TALE-mediated base editors are in use; each has its own pros and cons. The development of these base editors involves the fusion of CRISPR or TALE systems with the base editing module. CRISPR-Cas9 base editors include cytidine base editors and adenine base editors, resulting in C to T and A to G conversions, respectively. These Cas9 base editors have been reported in plants as well as humans for single-stranded DNA (ssDNA) base editing of nuclear genomes, failing to achieve significant desirable edits in the mitochondrial genome due to poor single-guide RNA delivery. To overcome this limitation, DdCBE and TALED have been reported for C to T and A to G conversions, respectively, with high editing efficiencies of mammalian mitochondria as well as plant chloroplast genomes.^4^^,^^5^^,^^6^^,^^7^ Incorporation of dsDNA-specific deaminase in these base editors (DdCBE and TALED), though, can mitigate the obstacle of editing organellar genomes but carries other setbacks like unintended mutations in the non-target strand of DNA.^8^ Thus, there is a need for a more precise base editing system that can overcome these limitations.

### CyDENT: A CRISPR-free strand-specific base editor

Recently, Hu et al. have introduced a new agent in this duel of TALEs and CRISPR, which is continuous since the advent of genome editing till today. They have presented a novel programmable CRISPR-free, all-protein-based, and strand-specific bespoke base editing system termed CyDENT array that tackles the constraints of both organellar genome editing and dsDNA-specific deaminase and could be an attractive alternative to the traditional base editors. Each module of this array is cloned along with a target genome localization signal into a separate vector for its localized activity in the target organelle, i.e., nucleus, mitochondria, or chloroplast. Moreover, the introduction of a mutation in any domain of FOKI dimer to develop FOKI nickase serves to confer the strand specificity to the CyDENT base editor.^9^ Authors have successfully achieved base editing using CyDENT with editing efficiency of up to 18% and 39% in plant nuclear and human mitochondrial genomes, respectively, with 95% target strand specificity. Base editing in the plant chloroplast genome has also been observed with CyDENT but with editing frequency as low as 1.67%, which could be attributed to the low transformation efficiency by PEG in rice protoplast. Moreover, they have evaluated different parameters, including fusion of the UGI (uracil DNA-glycosylase inhibitor) domain, fusion of a small peptide known as γb, the position of γb in the CyDENT array, and the introduction of different ssDNA-specific cytidine deaminases to improve the base editing efficiency of the CyDENT editor. The same protein modular array has been tested using adenine base editors (AdDENT) but with very low editing efficiencies, i.e., 0.90% and 2.10% in plant and human nuclear genomes, respectively. Conclusively, Hu et al. have envisaged the fact that modular protein assembly, making possible the choice of different exonucleases, deaminases, and position of additional fusion components, could help to customize and develop the CyDENT base editor with much higher precision and efficiency.^9^

The recent structural work has contributed to the knowledge of CRISPR-free base editing mechanisms. Xiang et al. came up with the first cryo-electron microscopy (cryo-EM) structures of the DddA-derived cytosine base editor (DdCBE), highlighting how its “lysine grips” and “hydrophobic trio” motifs indicate a limited editing window and drive the creation of the *WinPred* model for single-nucleotide precision for editing in mitochondrial DNA.^10^ These findings of DdCBE’s mechanistic framework highlight the significance of structure-guided engineering in boosting editing specificity and reducing off-target effects. Here, CyDENT can be seen as a conceptual and functional advancement, which has a modular, CRISPR-free, strand-specific platform with the ability to perform nuclear and organellar genome editing. By integrating design logic similar to the precision principles found in DdCBE, *CyDENT* delivers high-fidelity base editing beyond mitochondria, enabling the programmable manipulation of various genomic compartments in both plants and humans.^9^

Recent developments in the field of mitochondrial genome editing have increased the range of therapeutic options in the treatment of mitochondrial diseases through accurate manipulation of mutations in the mitochondrial genome. Comprehensive studies have pointed out the emergence of technologies such as mitoTALENs, DdCBE, and mitoBEs, which enable targeted repair or modulation of mitochondrial heteroplasmy to reduce pathogenic variants.^11^ Nevertheless, these platforms have major technical limitations, such as poor delivery into mitochondria, inability to control the size of the editing window, and the possibility of off-target deamination. In this context, *CyDENT* allows more precise and localized base conversions in both nuclear and organellar genomes, *broadening the scope* of mitochondrial genome engineering for safer and more programmable therapeutic interventions.

### Applications of CyDENT in human therapeutics and agriculture

**Figure 1:**
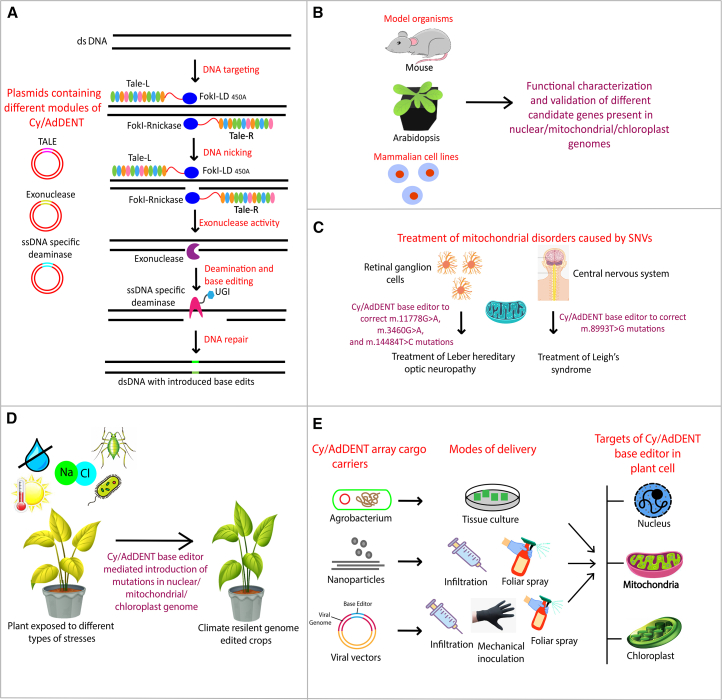
Schematics illustrating the mechanism of Cy/AdDENT base editor and its applications in human and plant biology (A) Each module of the Cy/AdDENT array (TALE, exonuclease, and ssDNA-specific deaminase; each having a target organelle localization signal) is cloned into a separate plasmid. TALE with a mutation in one member of the FokI dimer acts as a nickase and generates a nick at the target location. Exonuclease cleaves the nicked strand, deaminase acts on the exposed single strand of DNA, and a permanent base edit is produced at the target location. (B) The Cy/AdDENT base editor can be used to characterize and functionally validate the distinct genes in model organisms and mammalian cell lines. (C) Various mitochondrial disorders, including Leber hereditary optic neuropathy (LHON), mitochondrial encephalomyopathy, lactic acidosis, stroke-like syndrome (MELAS), and Leigh’s syndrome caused by SNVs can be treated by correcting the point mutations with the Cy/AdDENT base editor. (D) Cy/AdDENT base editor holds significant potential to develop climate-resilient genome-edited crops. (E) *Agrobacterium*, nanoparticles, and viral vectors can serve as a carrier of the Cy/AdDENT base editor array in plants. The deployment of viral vectors as a carrier results in transgene-free genome-edited plants. Each of the carriers delivers its cargo inside the cell where it gets transcribed, exported into the cytoplasm, and translated into proteins there. From the cytoplasm, these proteins then enter the target organelle (nucleus, chloroplast, or mitochondria), depending on the attached localization signal, to create base edits in the target genome.

This newly developed base editing system opens up new avenues for human therapeutics as well as plant genome engineering (Figure 1.). The strand-specific organellar genome base editing potential of CyDENT can be leveraged to characterize and functionally validate different candidate genes present in mitochondrial or chloroplast genomes, in addition to the nuclear genome in model organisms and human cell lines. Moreover, it can also be expedited to correct various human mitochondrial disorders, such as Leber hereditary optic neuropathy and Leigh’s syndrome, caused by single-nucleotide variations (SNVs) in the mitochondrial genome.^12^ In addition to this, it can offer a wide range of applications in crop improvement projects. We can enhance the photosynthetic activity by harnessing their ability to target the chloroplast genome, ultimately leading to improved crop yield. This novel base editor also carries a special significance for the development of climate-resilient crops by introducing point mutations in the target loci. In the current era of genome editing, nanoparticles and viral vectors have been reported to be efficient genome editing cargo carriers, overcoming the various limitations of traditional *Agrobacterium*-mediated plant transformation. Several reports have shown the delivery of cargo with the spray of nanovesicles^13^ and syringe infiltration of nanoparticles, which can be used to carry the CyDENT or AdDENT base editors to fine-tune the crops according to the increasing climatic variability in every season and close the yield gaps in the field. A large number of viral vectors, including tobacco rattle virus, potato virus X, and tomato spotted wilt virus, have been reported to efficiently deliver genome editing reagents.^14^ A recent study has documented the inoculation of viral vectors into plants by mechanical inoculation, foliar spray, and syringe infiltration.^15^ The independent module of each protein in the CyDENT array suggests that they can be easily delivered into target plant cells using viral vectors, overcoming the limitation of viral cargo capacity and producing transgene-free genome-edited plants, obviating any regulatory concerns. Moreover, instead of direct inoculation into the plant cell, viral vectors can be transformed into *Agrobacterium*, coated upon or encapsulated in nanoparticles to increase the penetration and editing efficiency.

Although significant base editing has been achieved using this novel base editor, one factor that can complicate its applicability is to custom-design the target-specific TALEs. Furthermore, engineering of deaminases for increasing the target base specificity to avoid bystander edits is also needed. Collectively, this base editing system expands the existing genome editing toolbox and holds promising potential in human disease modeling, therapeutics, and the development of climate-resilient crops. However, further research is needed to understand the mechanistic framework of this genome editing tool for new applications in plant and human biology.

## Declaration of interests

The authors declare no competing interests.
